# Genome-Wide Association Study for Grain Protein, Thousand Kernel Weight, and Normalized Difference Vegetation Index in Bread Wheat (*Triticum aestivum* L.)

**DOI:** 10.3390/genes14030637

**Published:** 2023-03-03

**Authors:** Gopalareddy Krishnappa, Hanif Khan, Hari Krishna, Narayana Bhat Devate, Satish Kumar, Chandra Nath Mishra, Om Parkash, Sachin Kumar, Monu Kumar, Harohalli Masthigowda Mamrutha, Gyanendra Pratap Singh, Gyanendra Singh

**Affiliations:** 1 ICAR-Indian Institute of Wheat and Barley Research, Karnal 132001, India; 2 ICAR-Sugarcane Breeding Institute, Coimbatore 641007, India; 3 ICAR-Indian Agricultural Research Institute, New Delhi 110012, India; 4 ICAR-Indian Agricultural Research Institute, Gauria Karma 825411, India; 5 ICAR-National Bureau of Plant Genetic Resources, New Delhi 110012, India

**Keywords:** wheat, GWAS, GPC, NDVI, candidate genes

## Abstract

Genomic regions governing grain protein content (GPC), 1000 kernel weight (TKW), and normalized difference vegetation index (NDVI) were studied in a set of 280 bread wheat genotypes. The genome-wide association (GWAS) panel was genotyped using a 35K Axiom array and phenotyped in three environments. A total of 26 marker-trait associations (MTAs) were detected on 18 chromosomes covering the A, B, and D subgenomes of bread wheat. The GPC showed the maximum MTAs (16), followed by NDVI (6), and TKW (4). A maximum of 10 MTAs was located on the B subgenome, whereas, 8 MTAs each were mapped on the A and D subgenomes. In silico analysis suggest that the SNPs were located on important putative candidate genes such as NAC domain superfamily, zinc finger RING-H2-type, aspartic peptidase domain, folylpolyglutamate synthase, serine/threonine-protein kinase LRK10, pentatricopeptide repeat, protein kinase-like domain superfamily, cytochrome P450, and expansin. These candidate genes were found to have different roles including regulation of stress tolerance, nutrient remobilization, protein accumulation, nitrogen utilization, photosynthesis, grain filling, mitochondrial function, and kernel development. The effects of newly identified MTAs will be validated in different genetic backgrounds for further utilization in marker-aided breeding.

## 1. Introduction

Wheat is one of the essential staple foods around the world. Wheat-based products are gaining increased demand because of changing dietary habits driven by urbanization along with industrialization. It is the main source of energy and starch and also provides a considerable quantity of protein, vitamins, dietary fibers, and phytochemicals that are beneficial or essential for health. Reduced secondary immunity due to protein energy malnutrition (PEM) is considered to be the most frequent cause of various diseases in human beings, and, in acute cases, clinically these are referred to as marasmus or kwashiorkor [1]. Further, severe PEM affects children’s cognitive development [2]. The protein concentration and composition are the two important determinants of both nutritional and end-use quality [3]. Gluten proteins (∼80%) are the major storage proteins that influence the baking process through flour’s functional properties. Grain protein content and hardness are the two key determinants of wheat grain quality to classify quality class in international trade and also to decide the suitability of the quality class to different types of end products [4].

The protein concentration in wheat is the result of genetic makeup, environmental effect, and genotype-environment interaction (GEI). GPC is a highly complex quantitative trait with substantial environmental (particularly nitrogen availability) and GEI effects. Several studies suggested the substantial effect of environment and GEI on GPC, TKW, and NDVI traits [5,6,7,8]. The process of protein enhancement is further complicated by the existence of trade-off between grain protein and yield in wheat. Therefore, crop improvement programs need to infuse more genetic diversity using wheat landraces and crop wild relatives [9]. High-grain protein concentration has been effectively transferred to modern cultivars through traditional plant breeding methods. High protein genes present in genetic resources like Atlas 50 and Atlas 66 have widely been utilized in breeding programs [10]. In addition, wild relatives have been utilized for a few high-protein genes in different breeding programs. For instance, in Israel, wild emmer, particularly accession FA15-3, is one of the most widely exploited germplasm for high grain protein, which can accumulate about 40% protein under adequate nitrogen fertilization [11]. The region controlling GPC is mapped on the 6BS chromosome and is designated as the *Gpc-B1* gene [12]. The designated gene through a NAC transcription factor, i.e., NAM-B1, has a pleiotropic effect, which enhances protein, iron, and zinc [13], due to faster senescence leading to remobilization of nutrients from source to sink [14].

The NDVI estimation provides the overall quantification of ground coverage along with the nitrogen status of the crop. It is an important physiological tool having high correlations with yield, biomass, and nitrogen content in wheat [15]. The NDVI could be used as a surrogate trait for the indirect assessment of leaf health for photosynthesis [16]. Therefore, both GPC and NDVI are related traits with much dependency on the genetic potential for nitrogen use efficiency. Although TKW in wheat has no nutritional importance by itself, but it has a concentration/dilution effect that influences the other nutrients including protein and micronutrient concentration. The simultaneous increase in grain yield coupled with reduced protein content was attributed to the dilution effect in wheat, which is demonstrated in many studies [17,18]. Thus, TKW is a key economic trait in crop improvement programs because of its role in the expression of grain yield and quality. While breeding for higher grain protein content, TKW always needs to be considered, as the shriveled grains always overestimate the protein content.

The quantitative traits like GPC, TKW, and NDVI need to be studied through genetic and molecular approaches for harnessing them through marker-assisted breeding (MAB). Further identifying linked molecular markers through QTL mapping would aid in the improvement of these polygenic traits [19,20]. The GWAS and quantitative trait loci (QTL) mapping are the two most commonly used approaches for understanding the genetics of quantitative traits. The conventional approach of QTL mapping depends on the genetic composition of bi-parental populations. A large number of QTLs have been identified in the last decade in wheat for the expression of GPC [21,22,23,24,25], TKW [26,27,28,29], and NDVI [30,31,32] through bi-parental-based conventional QTL mapping approach. However, the mapping resolution is very low in bi-parental population-based QTLs due to limited crossovers.

Alternatively, the linkage disequilibrium-based (LD) association mapping (AM) approach can enhance the genetic map resolution to a greater extent due to the representation of a wider gene pool and more recombination events in history [33]. The GWAS method detects non-random associations of markers distributed across the genome with the phenotype [34] and has extensively been utilized to identify marker-trait associations in crop plants [35]. The utilization of diverse lines that have accumulated more crossovers since their most recent progenitors diverged has greatly improved the QTL resolution in the GWAS mapping approach [36]. The GWAS approach overcomes the two general shortcomings, i.e., low allelic diversity and mapping resolution of bi-parental studies [37].

However, the control of the false positive rate in GWAS due to population structure and family association is one of the main limitations [38]. To reduce the false positives, a statistical package of BLINK is highly useful, as it eliminates the basic assumption of equal distribution of causal genes throughout the genome, and the statistical power is better when compared with other available GWAS models such as SUPER and FarmCPU [39].

Through the GWAS approach, many marker-trait linkages were found for GPC [40,41,42], TKW [43,44,45,46], and NDVI [47,48,49,50,51] in wheat using a GWAS panel with a diverse set of genotypes. Several MTAs/QTLs have been mapped on all three subgenomes of wheat; however, more mapping studies are required as the saturation point may not be reached [52]. Further, these traits are environmentally sensitive, hence, detection and validation of consistent MTAs in multi-location or multi-year studies are important to use in marker-aided breeding. Moreover, hexaploid wheat has three subgenomes with a size of ~17 Gb [53], with the limited characterization of LD decay. Thus, further studies are required to understand the genetic basis and devise marker-based breeding approach to further complement conventional breeding efforts. The present investigation aims to identify MTAs related to GPC, TKW, and NDVI in a diverse set of bread wheat genotypes in multi-environments following the GWAS method and putative candidate genes associated with the SNPs.

## 2. Materials and Methods

### 2.1. Genotypes and Field Experiments

**Figure 1:**
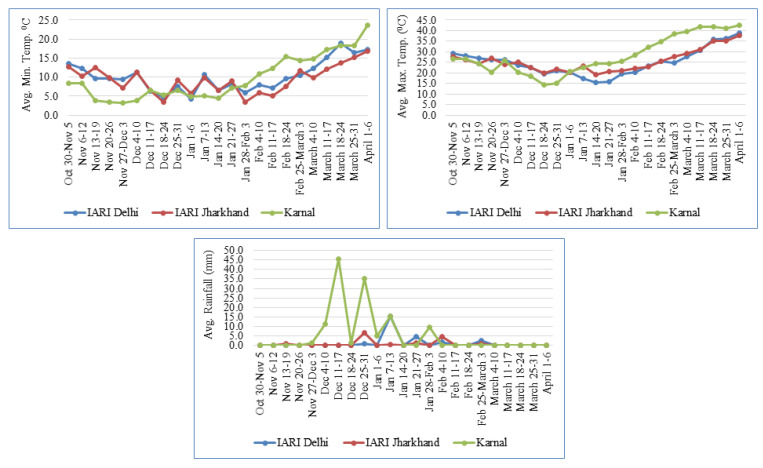
Weather parameters of the experimental sites during crop season 2021–2022.

A set of 280 diverse bread wheat genotypes, including advanced elite genotypes and commercial varieties, was used for the mapping study. The details of the genotypes used in the study are given in Table S1. The set of genotypes was evaluated in three different environments, i.e., E1-ICAR-Indian Agricultural Research Institute, New Delhi; E2- ICAR-Indian Agricultural Research Institute, Jharkhand; and E3- ICAR-Indian Institute of Wheat and Barley, Karnal. The weather parameters of the experimental sites during crop season 2021–22 is illustrated in Figure 1. The experiment was conducted under irrigated conditions, and planting was done from 1st to 15th November at all the locations during the year 2021–2022 Rabi (winter) season. The recommended dose of NPK in the ratio of 150:60:40 kg/ha was applied as urea and diammonium phosphate (DAP) for nitrogen, DAP for phosphorus, and muriate of potash for potassium. Biotic stresses were effectively controlled by the fungicide (tebuconazole 25% EC), pesticide (imidacloprid 30.5 SC), and pre-emergence herbicide (pendimethalin 30% EC). An augmented block design was used, in which checks (DBW 187, MACS 6222, WH 1124, and WH 1142) were replicated in each block of two rows of two-meter length. 

### 2.2. Data Recording and Statistical Analysis 

Phenotyping of 280 genotypes for GPC, TKW, and NDVI was done at three locations. The GPC was recorded with the infra-red transmittance-based instrument Infratech 1125, and the estimated readings were expressed at a 12.0% moisture level. A random sample of 1000 grains was counted, and the weight has been measured in an electric weighing machine. NDVI was recorded at the maximum vegetative stage (Zadok’s scale 41) using a handheld crop sensor, i.e., GreenSeeker (Trimble industries, Inc., Westminster, CO, USA), which was held 50 cm above the canopy facing the center of the plot to record the NDVI. Approximately, 3–4 NDVI readings/plot were recorded, and the mean represents the NDVI reading for that particular plot. The data for various genetic parameters were analyzed using the R package “augmented RCBD” [54].

### 2.3. Genotyping and Quality Control (QC) 

The genomic DNA was extracted using the cetyltrimethylammonium bromide (CTAB) method [55]. The pure seeds of each genotype were sown in a plastic tray with separate chambers, and the leaf samples were collected from 21-day-old seedlings from each genotype. The collected leaf samples were thoroughly washed with distilled water and dried on blotting paper. The dried leaf samples were cut into 1.5–2.5 cm length and kept in a mortar and pestle. The leaf sample was finely ground and transferred into a 2.0 mL Eppendorf tube, added with 700 μL pre-warmed 2% CTAB extraction buffer along with 0.2 vol% β-mercaptoethanol, and incubated in a water bath at 65 °C for 45 min. A total of 750 μL of chloroform/iso-amayl alcohol in the ratio of 24:1 was put into the tube and shaken thoroughly. The mixture in a tube was centrifuged at 12,000 rpm for 12 min, and the resulting supernatant was collected in a 1.5 mL tube. Later on, 700 μL cold iso-propanol was added and shaken slowly by inverting the Eppendorf tube. The tubes were kept under freezing conditions for 2 h at −20 °C and subsequently centrifuged at 12,000 rpm for 10 min, which results in DNA pellet. The DNA was treated with RNaseA, and the concentration was determined in a NanoDrop spectrophotometer. The genotyping of 280 genotypes was done using Axiom Wheat Breeder’s genotyping array (Affymetrix, Santa Clara, CA, USA) consisting of 35,143 genome-wide SNPs. Stringent quality control was applied through the removal of monomorphic markers, markers with minor allele frequency (MAF) of 20%, and heterozygote frequency of >25.0%. A total of 14,790 curated markers were further utilized for the GWAS study.

### 2.4. Population Statistics and GWAS 

Pairwise LD values (r2) were calculated using Analysis by aSSociation Evolution and Linkage (TASSEL) version 5.0 [56]. The LD block size of the whole genome, as well as individual subgenomes was calculated by fixing the r^2^ threshold at half LD decay. The PCA and kinship association were estimated using GAPIT [57]. Phenotypic data of GPC, TKW, and NDVI of the GWAS panel and corresponding genotypic data were used in GWAS analysis. Significant MTAs were detected through BLINK (Bayesian-information and linkage disequilibrium iteratively nested keyway) model [39] implemented in Genome Association and Prediction Integrated Tool (GAPIT) version 3.0 [58] in the R software package. The SNPs with *p* ≤ 0.0001 were considered significantly associated, and R^2^ reflects the percent phenotypic variation (PVE).

### 2.5. In Silico Analysis 

The sequence information of the significant SNPs was used to search for putative candidate genes with BLAST using default parameters in the Ensemble Plants database (http://plants.ensembl.org/index.html (accessed on 23 December 2022)) of the bread wheat genome (IWGSC (RefSeq v1.0)). The genes located in the overlapping and within the region of 0.1 Mb intervals flanking either side of the linked marker were recorded as putative candidate genes. The role of the detected genes in the regulation of GPC, TKW, and NDVI was also determined by comparing with the earlier reports in both wheat and other crop plants.

## 3. Results 

### 3.1. Variability, Heritability, and Correlation

**Figure 2:**
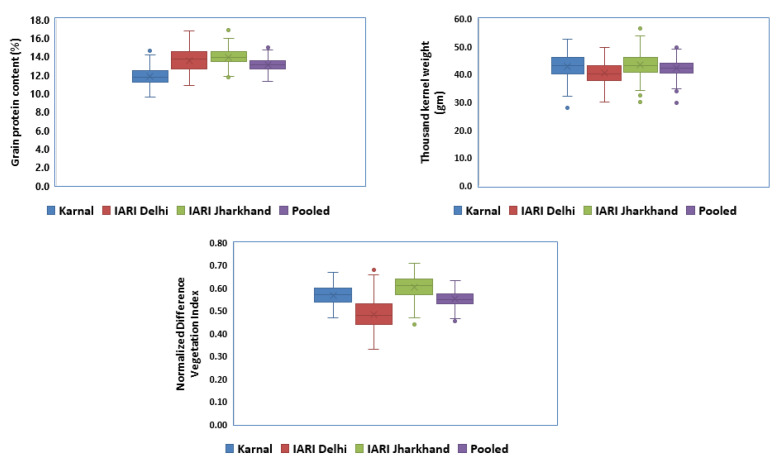
Boxplots of GPC, TKW, and NDVI in a set of 280 genotypes.

The genetic parameters of 280 genotypes given in Table 1, which exhibited a large range of variability across the environments for GPC, TKW, and NDVI, ranging from 09.59–16.71%, 28.38–55.98 gm, and 0.32–0.71, respectively. The percent CV was less than 8.0% in all the environments for all three traits, which ranged from 3.42–5.47% (GPC), 2.37–3.85% (TKW), and 6.58–7.65% (NDVI). Out of all the studied environments, E3 was found to be a relatively higher CV for all the traits. The trend of broad sense heritability of both GPC and NDVI are similar and lower than TKW, which recorded more than 80.0% in all the environments. The trait’s mean values are presented in Figure 2 as boxplots. All three traits recorded comparatively higher trait mean values in the E3 environment compared to other environments. The lowest trait mean values for GPC were recorded at E3, whereas, the lowest mean values for TKW and NDV were recorded at E2.

**Figure 3:**
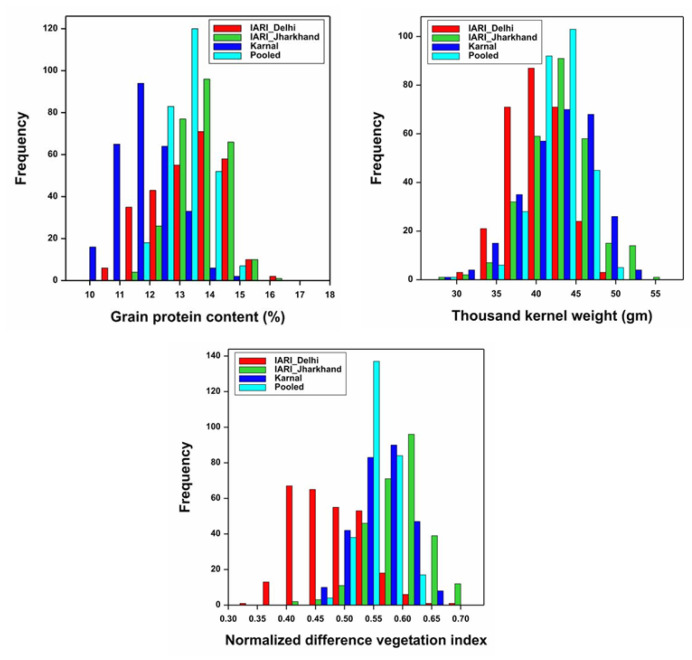
Frequency distribution in GWAS panel.

**Figure 4:**
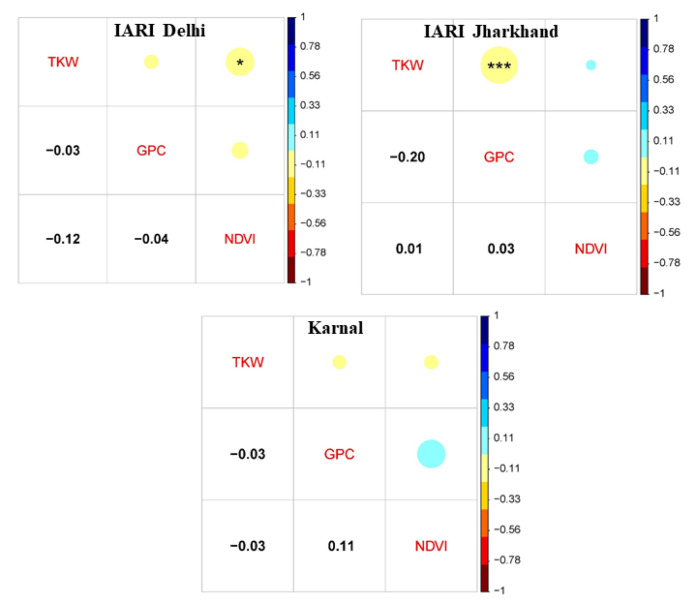
Phenotypic correlation coefficients for GPC, TKW, and NDVI in GWAS panel. * Significant at *p* < 0.05, *** Significant at *p* < 0.001. GPC: grain protein content; TKW: 1000 kernel weight; NDVI: normalized difference vegetation index.

The frequency distribution of GPC, TKW, and NDVI in a set of 280 genotypes tested at E1–E3 during 2021–2022 is illustrated in Figure 3. The continuous frequency distribution was observed for GPC, TKW, and NDVI. Pearson’s correlation coefficient (r^2^) was estimated and illustrated in Figure 4. The direction of the correlation between GPC and TKW was similar in all three environments, and it was negatively associated. Further, a significant and strong negative correlation between GPC and TKW was observed in E2. None of the environments recorded a significant correlation between GPC and NDVI; however, the direction of the correlation was positive in E2 and E3, whereas, it was negative in E1. Similarly, the association between TKW and NDVI was negative and significant at E1, and also the direction of the association was similar in E3. However, the direction of the association was positive in E2.

### 3.2. Marker Statistics 

**Figure 5:**
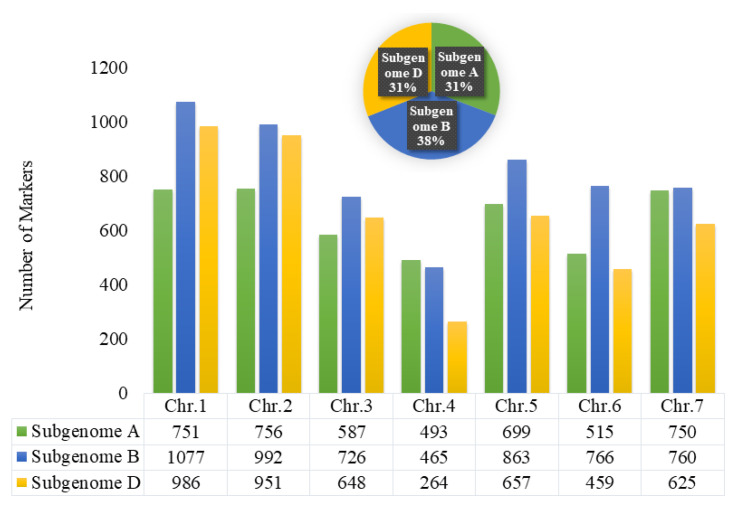
Chromosome-wise distribution of polymorphic markers; pie-chart represents the percentage of marker distribution on subgenome A, B and D.

The genome-wide distribution of SNPs is illustrated in Figure 5. After a thorough quality check on the 35K SNP array, 14,790 high-quality markers were chosen. These markers that qualify for quality control are further utilized to identify MTAs through GWAS analysis. The subgenome-wise distribution of SNPs was highest with 5649 on subgenome B, whereas, the other two subgenomes were represented similarly with 4590 (subgenome D) and 4551 (subgenome A). Similarly, chromosome-wise maximum SNPs of 1077 were identified on 1B chromosome, whereas, the lowest number of 264 SNPs were identified on the 4D chromosome.

### 3.3. Population Structure and LD

**Figure 6:**
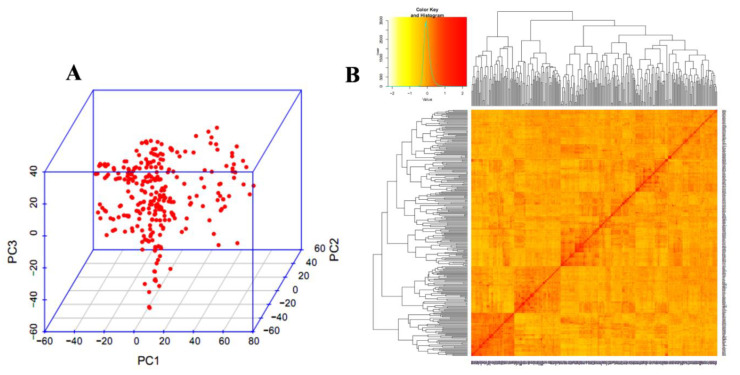
Population structure of 280 genotypes. (**A**) Principal component analysis (**B**) Heat map.

The PCA and kinship relationship of the GWAS panel is illustrated in Figure 6, which reveals the absence of clear-cut sub-groups. The LD was calculated by using the squared correlation co-efficient (r^2^) of all the SNPs. The LD decay was rapid with 3.6 cM in A subgenome, followed by 5.2 cM in D subgenome and 5.7 cm in B subgenome, whereas, the whole genome LD decay was 4.9 cM.

### 3.4. Genome-Wide Association Studies

**Figure 7:**
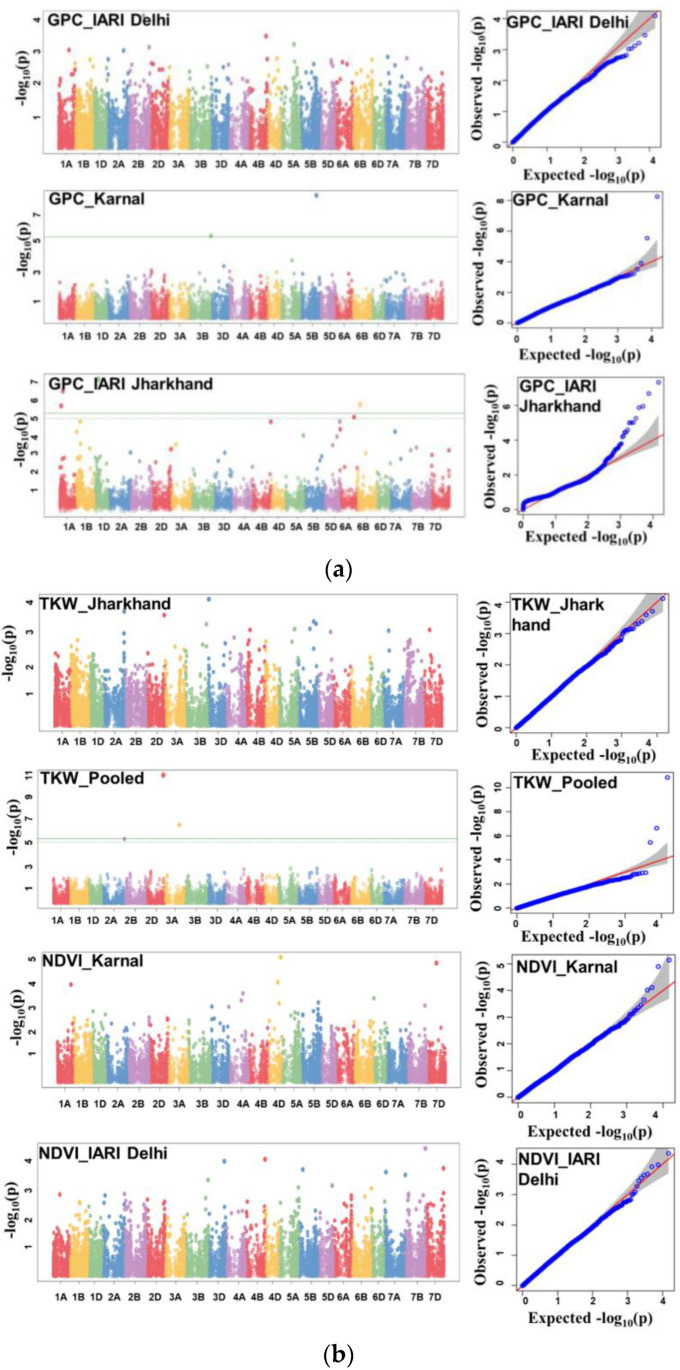
(**a**) Manhattan and respective Q–Q plots for grain protein content in GWAS panel; (**b**) Manhattan and respective Q–Q plots for thousand kernel weight, and normalized difference vegetative index in GWAS panel.

A set of 26 significant MTAs was detected including 16 for GPC, 4 for TKW, and 6 for NDVI (Table 2). The details of the detected MTAs are presented in Table 2 and illustrated as Manhattan plots in Figure 7a,b. The Q–Q plots depicting the observed associations of SNPs of GPC, TKW, and NDVI compared to the expected associations after accounting for population structure are presented in Figure 7a,b.

A set of 16 significant MTAs was detected for GPC in E1, E2, and E3 on 1A, 1B, 1D, 2B, 3B, 4B, 5A, 5B, 5D, 6A, 6B, and 7A and PVE ranged from 6.2% (*AX-94749397*) to 11.4% (*AX-95248629*). Out of 16 MTAs, *AX-95248629* (5B), *AX-94746929*(3B), *AX-94714023* (2B), and *AX-94520919* (5D) explained more than 10.0% PVE, which were located at 580.4 Mb, 800.9 Mb, 536.3 Mb, and 550.1 Mb, respectively. The highest number of MTAs (13 nos.) were identified in E2 for GPC. The highest number of seven MTAs, i.e., *AX-94714023* (2B), *AX-94412218* (6B), *AX-94770504* (4B), *AX-95082115* (1B), *AX-94749397* (1B), *AX-95248629* (5B), and *AX-94746929* (3B) were identified on B subgenome and located at 536.3 Mb, 100.2 Mb, 667.6 Mb, 144.1 Mb, 16.4 Mb, 58.4 Mb, and 800.9 Mb, respectively. PVE ranged from 6.2% (*AX-94749397*) to 11.4% (*AX-95248629*). Similarly, six MTAs, i.e., *AX-94825050* (1A), *AX-95107750* (1A), *AX-94384140* (5A), *AX-94537786* (6A), *AX-95186193* (6A), and *AX-95199688* (7A) were identified on A subgenome and located at 531.8 Mb, 112.9 Mb, 659.1 Mb, 501.1 Mb, 33.1 Mb, and 171.3 Mb, respectively. The PVE ranged from 6.6% (*AX-95107750*) to 7.7% (*AX-94825050*). However, only three MTAs, i.e., *AX-94675928* (1D), *AX-94520919* (5D), and *AX-94617912* (5D), were mapped and located at 112.3 Mb, 550.1 Mb, and 450.6 Mb, respectively. The PVE ranged from 6.3% (*AX-94617912*) to 10.1% (*AX-94520919*).

For TKW, one major MTA (*AX-94651901)* on 3D was detected in E2 at 40.1 Mb and explained 13.8% PVE. The remaining three MTAs, i.e., *AX-94454052* (2D), *AX-94861851* (3A), and *AX-95194336* (2B), were detected for pooled mean, located at 617.0 Mb, 544.3 Mb, and 96.2 Mb, respectively with PVE ranging from 8.7% (*AX-95194336*) to 13.4% (*AX-94454052*). For NDVI, six MTAs, viz., *AX-94826552* (7B), *AX-95111632* (4B), *AX-94978133* (4D), *AX-94493107*(7D), *AX-94736370* (4D), *AX-95006755* (1A), were identified and located at 717.2 Mb, 667.8 Mb, 465.7 Mb, 306.7 Mb, 359.1 Mb, and 485.3 Mb, respectively with the explained PVE ranging from 6.2% (*AX-95006755*) to 12.1% (*AX-94826552*).

### 3.5. Putative Candidate Genes Associated with MTAs

The SNPs linked to GPC, TKW, and NDVI were further utilized to detect the putative genes using the annotated wheat reference sequence (Wheat Chinese Spring IWGSC Ref Seq v2.1 genome assembly (2021)) and are given in Table 3. SNPs, i.e., *AX-94537786, AX-94520919, AX-94770504, AX-95199688*, and *AX-95107750*, associated with GPC were found to encode lateral organ boundaries, LOB (TraesCS1A02G111700), Zinc finger, RING-H2-type (TraesCS6A02G274400), Zinc finger, RING-H2-type (TraesCS6A02G274400),NAC domain (TraesCS5D02G537600), Folylpolyglutamate synthase (TraesCS4B02G392600), and Aspartic peptidase domain (TraesCS7A02G208600), respectively. One SNP, i.e., *AX-94651901* associated with TKW was found to encode serine/threonine-protein kinase LRK10-like (TraesCS3D02G011300) and Pentatricopeptide repeat (TraesCS3D02G011200). Similarly, *AX-94454052* associated with TKW encodes protein kinase-like domain superfamily (TraesCS2D02G530900). In addition, two SNPs, i.e., *AX-95111632* and *AX-94978133* associated with NDVI, were found to encode Cytochrome P450 (TraesCS4B02G393700) and Expansin (TraesCS4D02G296100).

## 4. Discussion

Although yield enhancement has been the main focus of crop improvement programs across the globe for a long time, wheat quality enhancement is gaining importance only in the recent past. Wheat improvement for quality is a tedious, expensive, and time-taking process, which makes quality improvement programs slow and protracted. Further, yield and quality enhancement in wheat was mostly phenotype-based selection through conventional breeding for many years. However, genotype-based approaches can complement conventional methods in cultivar development programs. Moreover, recent efforts that led to the sequencing of the wheat genome could further enhance the potential of marker-based breeding in wheat. Several MTAs/QTLs were detected for various economic traits in wheat. However, further genetic studies are suggested using different germplasm or populations as mapping has not reached a saturation level [52]. Further, hexaploid wheat has three subgenomes with a large genome size of ~17 Gb, and there is always a possibility to map new QTLs/MTAs for quality traits. In addition, ample genetic diversity is present in the unexplored gene bank accessions and elite breeding materials, which make suitable candidates to dissect the genetic basis and to identify novel MTAs through GWAS analysis.

### 4.1. Variability, Correlation, and GEI

The expression of GPC, TKW, and NDVI has been greatly influenced by the effects of the environment and GEI. GPC was relatively more environment-sensitive, whereas, TKW was a largely stable trait. Significant effects of environment and GEI have been described in earlier reports [5,6,7,8,79]. The GWAS panel has been evaluated in multi-environments, as GEI is an important factor to identify environment-specific and consistent QTLs. The trait’s environmental sensitivity was also reflected in the trend of broad sense heritability, as TKW recorded high heritability as compared to TKW and NDVI. Similarly, TKW has recorded the lowest percent CV and highest GCV compared to the other two traits.

The negative association between TKW and GPC observed in the current study was also reported previously in several reports [42,80]. This well-established negative correlation between GPC and TKW was partly explained by the dilution effect [17,18]. This negative association may also be attributed to nutrition (particularly nitrogen) competition between TKW and GPC. Although the correlation between GPC and TKW was not significant, the direction of association was positive. The positive and significant association between GPC and NDVI was also observed in the previous studies [81]. However, the correlation between TKW and NDVI was significant and negative. Previously a significant and negative correlation between TKW and NDVI was also reported [49].

### 4.2. Linkage Disequilibrium

The PCA analysis of the high-quality SNPs that exhibited allelic frequency was evenly distributed without any separate sub-populations. Allelic frequency of equal distribution was obtained through the careful selection of elite breeding lines for different agro-ecological zones. Wheat being a self-pollinated crop generally has a larger LD block size and, hence, slowly decays [82], compared to cross-pollinated crop plants like maize, where the LD decay is rapid [83]. QTL mapping resolution may be reduced due to presence of large LD blocks and vice versa [84]. The LD decay distance of ~3 cM of A subgenome is shorter than the B and D subgenomes, which have a decay distance of ~5 cM. A similar LD decay pattern was also recorded previously in wheat GWAS studies [44,84,85]. The LD among the populations may vary due to various factors including population size, non-random mating, random genetic drift, selection, admixtures, mutation, pollination pattern, and recombination frequency [86,87].

### 4.3. MTAs

A set of 26 MTAs was identified for GPC (16), TKW (4), and NDVI (6). A maximum of 10 MTAs were mapped on the B subgenome, whereas, 8 MTAs each were mapped on the A and D subgenomes. The pattern of subgenome-wise marker distribution is also similar, as maximum markers were located on subgenome B, and an approximately similar number of markers were mapped on A and D subgenomes. In earlier studies also, a similar pattern of QTL and marker distribution among the subgenomes for grain-quality traits was reported [21,26]. Krishnappa et al. [22] studied a RIL population wherein none of the QTL was identified on the D subgenome due to a very less distribution of markers; however, the enrichment of the D genome with additional SNP markers in the same mapping population has significantly increased the power of QTL identification. Therefore, marker frequency and distribution along with the type and size of the mapping population are important determinants of QTL mapping.

The total of 16 MTAs detected for GPC on different chromosomes in the present study is new, as the previously reported MTAs/QTLs were identified at different locations of the same chromosomes. In previous studies, MTAs for GPC were also reported in different mapping populations on 1A [25,27,46,88], 1B [24,25,27,80,87,88], 1D [46], 2B [24,25,27,41,49], 3B [24,41,80], 3D [25,27,41,46,88,89], 5A [24,88], 5B [41], 5D [46,80,90], 6A [27,88,89,90], 7A [22,41], and 7B [41]. Similarly, four MTAs were identified on 3D, 2D, 3A, and 2B for TKW. MTAs for TKW on the same chromosomes in different mapping populations were also identified in earlier reports on 1B [27], 2B [28,91], 2D [25,80,91,92,93], 3A [88,93,94,95] at different chromosomal locations. Cabral et al. [27] identified a QTL, i.e., *QGwt.crc-2B-2*, on the 2B chromosome, located at a confidence interval of 92.9–96.0 cM, which was similar to the MTA (*AX-95194336*) detected in the current study on the same chromosome at 96.2 Mb. For NDVI, six MTAs were mapped on 1A, 4B, 4D, 7B, and 7D. Earlier studies also reported the MTAs for NDVI on the same chromosomes like 1A and 4B [31] and 4B and 4D [32].

### 4.4. Putative Candidate Genes 

Through BLAST search, several putative candidate genes underlying MTAs for GPC, TKW, and NDVI were identified (Table 3). The MTAs identified on different chromosomes of wheat are present in the gene coding regions associated with different transcription factors, transmembrane proteins, zinc finger superfamilies, etc. For instance, *AX-94520919* linked to GPC encodes NAC domain (TraesCS5D02G537600) genes, which regulate protein accumulation in wheat grains. A NAC transcription factor (*NAM-B1*) that enhances nutrient redistribution from source to sink and accelerates senescence is encoded by the ancestral wild wheat allele [14]. Another transcription factor, i.e., the *OsNAC*-like transcription factor, is reported to regulate seed-storage protein concentration in rice [62]. An NAC transcription factor (*HvNAM1*) controls anthesis time, senescence, and grain protein content in barley [63]. NAM proteins control the movement of nitrogen, zinc, and iron from vegetative tissues to developing grains in wheat [64]. NAC transcription factors enhance the acceleration of leaf senescence, and thereby remobilize iron and zinc to seeds in rice [65]. An SNP, i.e., *AX-94770504* linked to GPC, encodes folylpolyglutamate synthase (TraesCS4B02G392600) found to have a role in nitrogen utilization. In the early seedling development stage, the gene for mitochondrial folylpolyglutamate synthetase regulates nitrogen utilization in Arabidopsis [66]. Similarly, another SNP, i.e., AX-94537786 associated with GPC, encodes zinc finger, RING-H2-type (TraesCS6A02G274400), that controls the protein accumulation in wheat. In rice, the CCCH-type zinc finger protein (*OsGZF1*) regulates the *GluB-1* promoter, a seed storage protein, and controls the accumulation of glutelin protein during grain development [60]. The C2H2 zinc finger family transcription factor regulated grain-related traits in maize [61]. Further, *AX-95199688* associated with GPC encodes the aspartic peptidase domain (TraesCS7A02G208600) and was found to have a role in gluten breakdown. Gluten aspartic proteinase (GlAP 2) is associated with gluten breakdown in wheat [67].

Few putative candidate genes were also identified for TKW; for example, one SNP, i.e., *AX-94651901*, encodes serine/threonine-protein kinase LRK10-like (TraesCS3D02G011300), which has a role in grain weight regulation. A pentatricopeptide repeat protein that influences photosynthesis and grain filling is encoded by the kernel size-related QTL (*qKW9I*) [69]. The mitochondrion targeted pentatricopeptide repeat 5 regulates endosperm development in rice [70]. Two important pentatricopeptide repeat genes (*GRMZM2G353195* and *GRMZM2G141202*) are regarded as key candidate genes associated with maize kernel-related traits, including thousand kernel weight [71]. Pentatricopeptide repeat protein DEK45 [72], PPR18 [73], and ZmSMK9 [74] are required for mitochondrial function and kernel development in maize. The same SNP also encodes serine/threonine-protein kinase LRK10-like (TraesCS3D02G011300). The serine/threonine protein kinase encoding gene KERNEL NUMBER PER ROW6 (KNR6) regulates kernel number and ear length [68]. Another SNP, i.e., *AX-94454052*, encodes the protein kinase-like domain superfamily (TraesCS2D02G530900). *OstMAPKKK5* controls plant height and yield in rice [75]. The wheat protein kinase gene *TaSnRK2.95A* has a role in the regulation of high thousand kernel weight and grains per spike [76]. For NDVI, two putative candidate genes, i.e., cytochrome P450 (TraesCS4B02G393700) and expansin (TraesCS4D02G296100) were identified. The expansin regulates grain size in wheat [77]. In transgenic tobacco plants, the wheat expansin gene (TaEXPA2) increased drought tolerance [78].

## 5. Conclusions

The study with a set of 280 diverse bread-wheat genotypes revealed that GPC, TKW, and NDVI are quantitative traits. The negative association of GPC and TKW suggests that there is a trade-off between grain protein content and grain weight. However, GPC and NDVI are positively correlated, as both these traits are much influenced by the soil nitrogen status. A total of 26 MTAs, including 16 for GPC, six for NDVI, and four for TKW, were identified. Several putative candidate genes encoding main functions such as zinc, iron, and protein remobilization, increased nitrogen use efficiency, photosynthesis regulation, endosperm development, mitochondrial function, and stress tolerance are reported. Further, functional characterization of these putative genes to understand their role in wheat growth and development is envisaged.

## Figures and Tables

**Table 1:** Genetic parameters of GPC, TKW, and NDVI.

Trait	Env.	Mean ± SD	Range	CV (%)	LSD	*h^2^*BS	GCV	ECV
GPC (%)	E1	13.5 ± 1.18	10.81–16.71	4.77	1.94	70.28	7.32	4.76
	E2	13.9 ± 0.84	11.88–16.62	5.47	2.29	68.53	6.61	5.47
	E3	11.8 ± 0.91	09.59–14.81	3.42	1.23	72.80	6.96	3.46
TKW (gm)	E1	40.55 ± 0.21	31.01–50.41	3.14	3.83	86.56	7.95	3.13
	E2	43.36 ± 0.26	29.48–55.98	3.85	5.02	84.15	8.85	3.84
	E3	42.81 ± 0.26	28.38–52.98	2.37	3.05	94.64	9.95	2.37
NDVI	E1	0.49 ± 0.06	0.32–0.69	6.58	0.10	72.69	9.75	6.59
	E2	0.60 ± 0.05	0.44–0.71	7.65	0.14	68.18	7.28	7.64
	E3	0.57 ± 0.04	0.46–0.68	7.20	0.12	70.95	6.53	7.21

E1: IARI Delhi; E2: IARI Jharkhand; E3: Karnal; SD: standard deviation; CV: coefficient of variation; *h^2^*BS: broad sense heritability; GCV: genotypic coefficient of variability; ECV: environmental coefficient of variability.

**Table 2:** MTAs for grain protein, 1000 kernel weight, and normalized difference vegetation index.

Trait	Environment	SNPs	Chr.	Position	*p* Value	PVE (%)
Grain protein content (%)	E1	*AX-94714023*	2B	536316470	8.12 × 10^−5^	10.2
	E2	*AX-95107750*	1A	112941690	2.09 × 10^−7^	6.6
		*AX-94825050*	1A	53188500	1.35 × 10^−6^	7.7
		*AX-95082115*	1B	144122241	1.00 × 10^−5^	7.7
		*AX-94749397*	1B	16478742	3.53 × 10^−5^	6.2
		*AX-94675928*	1D	112354107	4.71 × 10^−8^	7.2
		*AX-94770504*	4B	667680308	9.97 × 10^−6^	7.0
		*AX-94384140*	5A	659165855	5.65 × 10^−5^	6.9
		*AX-94617912*	5D	450634975	6.54 × 10^−5^	6.3
		*AX-94520919*	5D	550185848	9.88 × 10^−6^	10.1
		*AX-94537786*	6A	501176793	5.53 × 10^−6^	7.7
		*AX-95186193*	6A	3311006	2.59 × 10^−5^	7.0
		*AX-94412218*	6B	100291191	1.14 × 10^−6^	7.9
		*AX-95199688*	7A	171387994	3.47 × 10^−5^	6.9
	E3	*AX-94746929*	3B	800933346	2.88 × 10^−6^	10.9
		*AX-95248629*	5B	580431598	5.61 × 10^−9^	11.4
Thousand kernel weight	E2	*AX-94651901*	3D	4012915	7.79 × 10^−5^	13.8
	Pooled	*AX-95194336*	2B	9620943	3.54 × 10^−6^	8.7
		*AX-94454052*	2D	617073435	1.41 × 10^−11^	13.4
		*AX-94861851*	3A	544385295	2.31 × 10^−7^	10.7
Normalized difference vegetation index	E1	*AX-95111632*	4B	667859119	1.06 × 10^−4^	10.6
		*AX-94826552*	7B	717202719	4.46 × 10^−5^	12.1
	E3	*AX-95006755*	1A	485355517	9.70 × 10^−5^	6.2
		*AX-94978133*	4D	465771817	7.36 × 10^−6^	10.1
		*AX-94736370*	4D	359118968	7.80 × 10^−5^	11.7
		*AX-94493107*	7D	306757146	1.28 × 10^−5^	11.5

E1: IARI Delhi; E2: IARI Jharkhand; E3: Karnal; PVE%: percent phenotypic variation explained.

**Table 3:** Putative candidate genes for GPC, TKW, and NDVI.

Trait	SNP ID	Position	Chr	Trace ID	Putative Candidate Genes	Function
GPC	*AX-95107750*	112941690	1A	TraesCS1A02G111700	Lateral organ boundaries, LOB	Stress tolerance in wheat [59]
	*AX-94537786*	501176793	6A	TraesCS6A02G274300	P-loop containing nucleoside triphosphate hydrolase	–
				TraesCS6A02G274400	Zinc finger, RING-H2-type	Regulates glutelin protein accumulation in Rice via controlling of *Glu B-1* promoter [60]. Regulation of grain-related traits in maize [61]
	*AX-94520919*	550185848	5D	TraesCS5D02G537600	NAC domain superfamily	Protein, iron, and zinc remobilization in wheat [14]. Regulation of seed-storage protein content in rice [62]. Controls percent grain protein in barley [63]. Remobilization of iron, zinc, and nitrogen from vegetative tissues to developing grains in wheat [64]. Iron and zinc remobilization to seeds in Rice [65]
	*AX-94770504*	667680308	4B	TraesCS4B02G392600	Folylpolyglutamate synthase	Nitrogen utilization in Arabidopsis [66]
	*AX-95199688*	171387994	7A	TraesCS7A02G208600	Aspartic peptidase domain	Gluten aspartic proteinase (GlAP 2) is associated with gluten breakdown in wheat [67]
				TraesCS7A02G208700	Aluminum-activated malate transporter	–
TKW	*AX-94651901*	4012915	3D	TraesCS3D02G011300	Serine/threonine-protein kinase LRK10-like	Regulates kernel number and ear length in Maize [68]
				TraesCS3D02G011200	Pentatricopeptide repeat	Controls photosynthesis and grain filling in maize [69]. Endosperm development in Rice [70]. Maize kernel-related traits including thousand kernel weight [71]. Pentatricopeptide repeat protein DEK45 [72], PPR18 [73] and ZmSMK9 [74] are required for mitochondrial function and kernel development in maize
	*AX-94454052*	617073435	2D	TraesCS2D02G530900	Protein kinase-like domain superfamily	OstMAPKKK5 controls plant height and yield in rice [75]. TaSnRK2.9-5A has a role in high TKW and grains per spike [76]
NDVI	*AX-95111632*	667859119	4B	TraesCS4B02G393700	Cytochrome P450	Regulates grain size in wheat [77]
	*AX-94978133*	465771817	4D	TraesCS4D02G296100	Expansin	TaEXPA2 regulates drought responsiveness in transgenic tobacco [78]

## Data Availability

Data is contained within the article.

## Supplementary Materials

The following supporting information can be downloaded at: https://www.mdpi.com/article/10.3390/genes14030637/s1, Table S1: List of genotypes.
